# P01-038 – QT and JT dispersion in children with FMF

**DOI:** 10.1186/1546-0096-11-S1-A42

**Published:** 2013-11-08

**Authors:** K Fidanci, A Kilic, M Gulgun, C Acikel, G Basbozkurt, E Demirkaya, F Gok

**Affiliations:** 1 Pediatric Cardiology, Gulhane Military Medical Faculty, Ankara, Turkey; 2 FMF Arthritis Vasculitis and Orphan Disease Research Center, Gulhane Military Medical Faculty, Ankara, Turkey; 3 Pediatric Rheumatology, Gulhane Military Medical Faculty, Ankara, Turkey

## Introduction

Familial Mediterranean Fever (FMF) is an autoimmune, autosomal recessive inherited disorder, and characterized by recurrent episodes of peritonitis, plöritis and arthritis. Patients with inflammatory disease are at increased risk of cardiovascular complications due to rhythm disorders. QT and JT dispersions are simple and non-invasive arrhythmogenic markers and can be used to assess the homogeneity of cardiac repolarization.

## Objectives

The aim of this study was to determine the risk of cardiac arrhythmias in patients with FMF by evaluating QT and JT dispersion.

## Methods

A total of 48 FMF patients who are in the attack-free period and use regular colchicine therapy (26 male, 22 female, 11.10 ± 3.42 years) and 31 healthy children (17 males, 14 females, 9.61 ± 2.83 years) were included in the study. The study group and the control group were evaluated with a standard 12-lead electrocardiography (ECG). QT, JT and RR distances were measured in both groups. The corrected QT (QTc) and corrected JT (JTc) were calculated. QT dispersion (QTcd) and JTd dispersion (JTcd) were determined.

## Results

There was no statistically significant difference was found between the study and control groups in terms of RR, QT, QT, QTcd, JT, JTc, JTd and JTcd measurements. QTc value is found to be higher in patients with FMF than the control group (412.15±21.45–393.58±35.18, t=2916, p=0.005), although the difference was statistically significant, the value is within normal limits (below 0.44).

## Conclusion

QTc value indicates the increased ventricular sensitivity and is an important marker of cardiovascular mortality. It has an important effect on sudden cardiac death and arrhythmia. In the ligths of these results, electrocardiographic monitoring may be useful in patients with FMF.

## Disclosure of interest

None declared.

